# Gastric adenomyoma in the stomach body: a case report

**DOI:** 10.1186/1752-1947-8-385

**Published:** 2014-11-24

**Authors:** Kyung Hwa Yoon, Dong Yeub Eun, Jae Hoon Kim, Soo Ok Lee, Hyun Soo Kim, Dong Wook Lee

**Affiliations:** 1 Division of Gastroenterology and Hepatology, Department of Internal Medicine, Daegu Fatima Hospital, 99 Ayangro, Dong-gu, Daegu, South Korea

**Keywords:** Adenomyoma, Stomach body, Endoscopic ultrasonography

## Abstract

**Introduction:**

Gastric adenomyoma is a rare benign tumor, known to occur in the antrum or pylorus of the stomach. To the best of our knowledge, this is the first case reported in the literature of a gastric adenomyoma in the stomach body, but not in the antrum.

**Case presentation:**

We report the case of a 79-year-old Korean woman with a gastric subepithelial lesion in the stomach body. The lesion was observed via endoscopy as a bulging mass in the stomach body with a cystic center. It was confirmed via endoscopic ultrasonography. A laparoscopic wedge resection was performed. A biopsy revealed epithelial and smooth muscle proliferation, and a final diagnosis of gastric adenomyoma was made.

**Conclusion:**

Gastric adenomyoma can occur in the stomach body and can be treated completely with surgery.

## Introduction

According to the World Health Organization’s histopathologic classification, gastric adenomyoma is a benign tumor of the stomach characterized by a smooth muscle stroma in the glandular structures of the cuboidal epithelium or columnar epithelium [1]. Magnus-Alsleben first reported this disease in 1903 [2]. Although gastric adenomyoma occurs most frequently in the gallbladder, a few cases in the stomach have been reported. Of the cases in the stomach, 85% occurred in the antrum and 15% occurred in the pylorus [3]. Furthermore, even though some patients may experience non-specific gastrointestinal symptoms such as epigastric pain or vomiting, most cases are asymptomatic [4,5]. In this report, we describe the case of a patient with a gastric subepithelial lesion in the stomach body that was discovered via endoscopic examination and diagnosed as a gastric adenomyoma. We also present a review of the related literature.

## Case presentation

**Figure 1:**
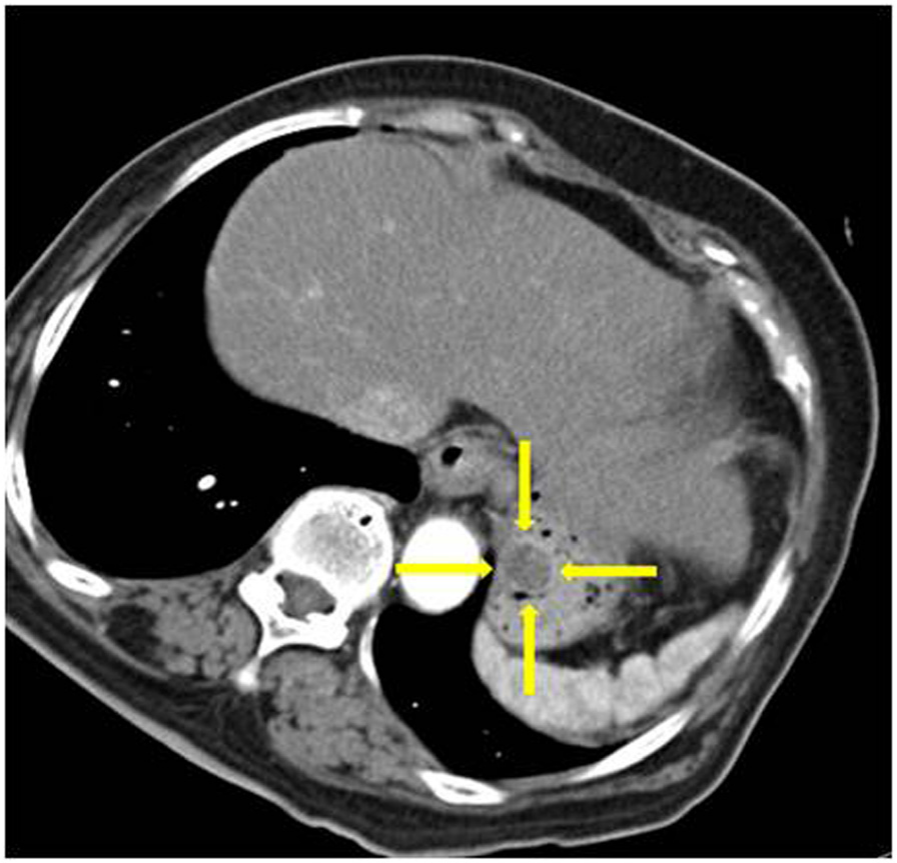
**Contrast-enhanced abdominal computed tomographic scan of the patient’s stomach.** Image shows a well-defined, heterogeneous, round mass in the stomach body (yellow arrows). Neither regional lymph node enlargement nor distant metastasis can be seen.

**Figure 2:**
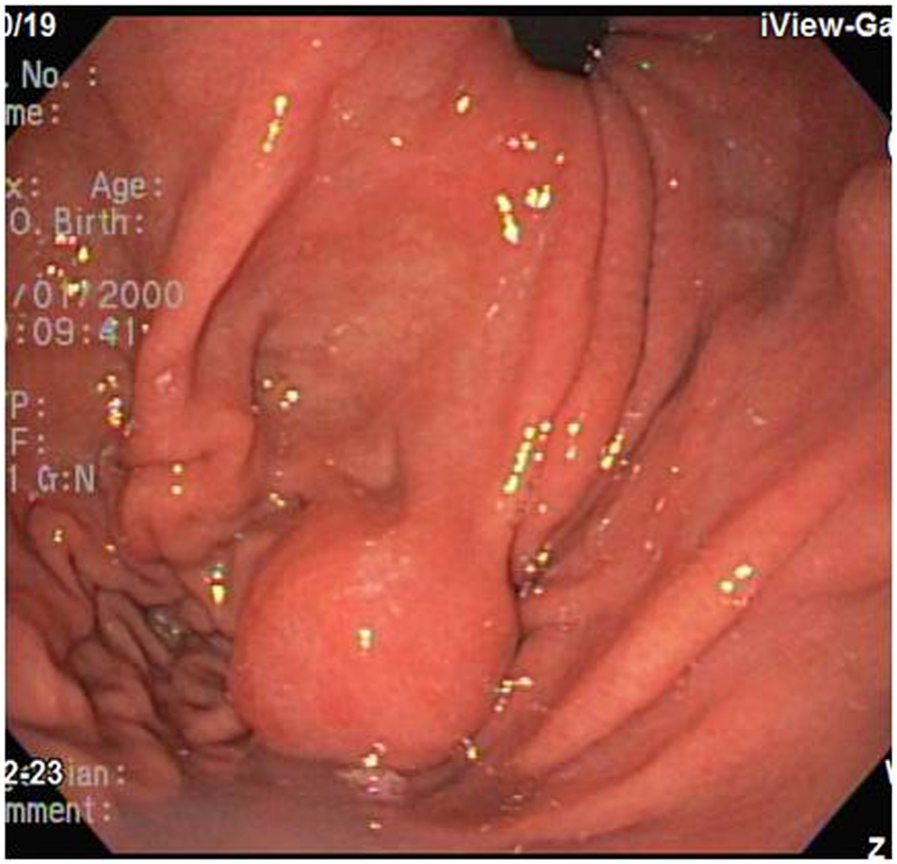
**Endoscopic findings.** A round subepithelial lesion covered with normal mucosa can be observed in the proximal body.

**Figure 3:**
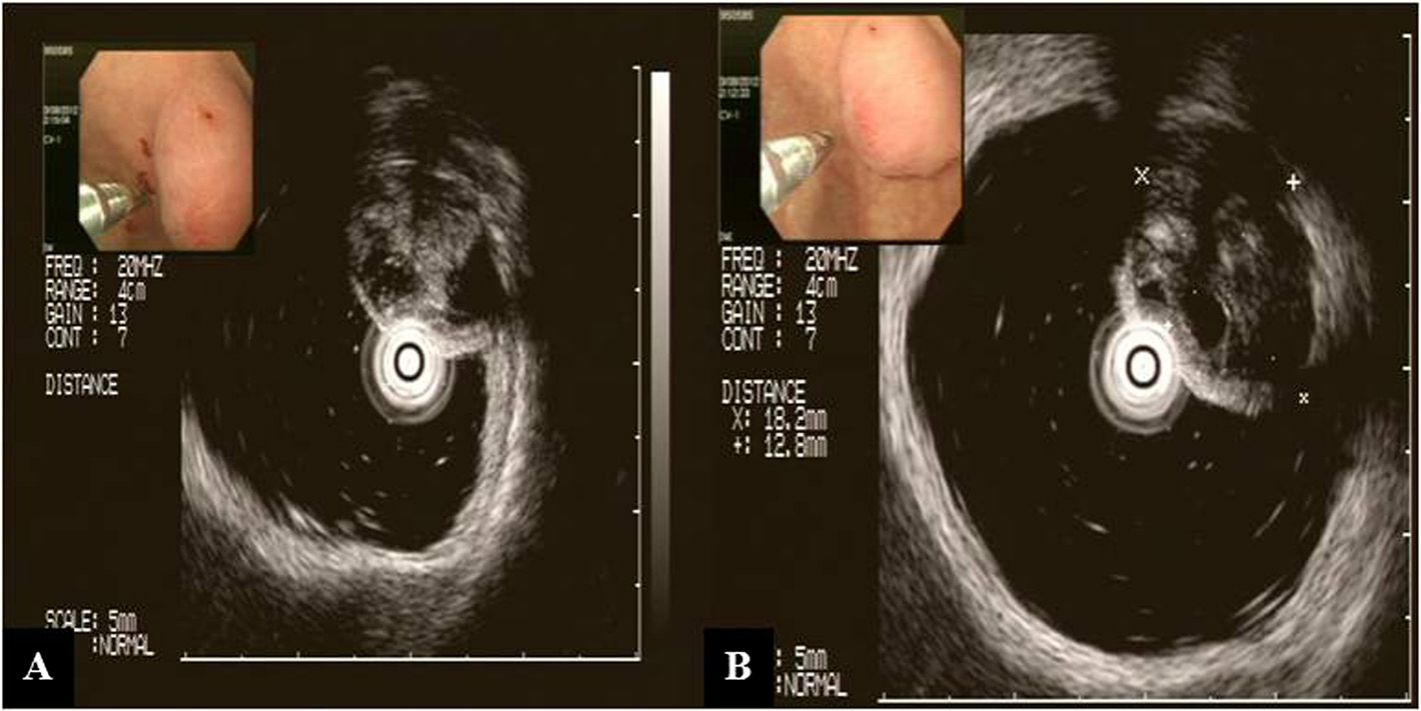
**Endoscopic ultrasonographic findings (A) and (B).** The lesion seems to originate from the third layer with heterogeneous echogenicity and an approximately 18mm focally cystic center.

**Figure 4:**
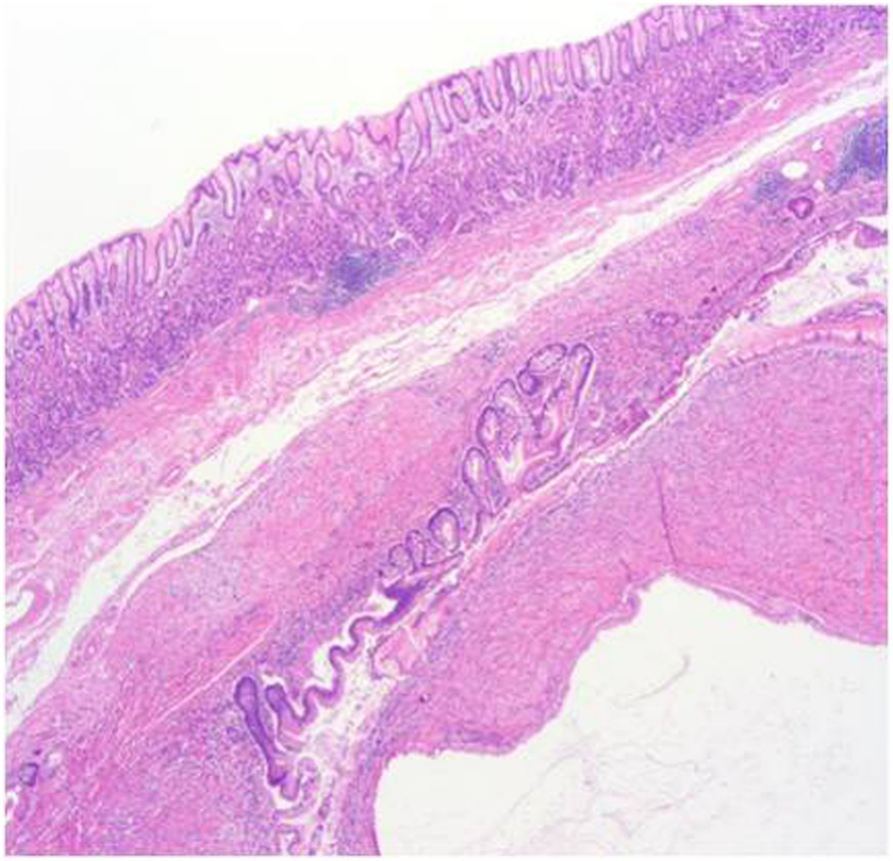
**Pathologic findings.** Hematoxylin and eosin staining shows fibromuscular stroma below the submucosal layer with a branched gland containing mucinous material.

A 79-year-old Korean woman was referred to our hospital for an incidentally detected subepithelial lesion in the stomach. Her medical history and alcohol intake, smoking history, and recent drug use were unremarkable. A physical examination did not reveal significant abnormalities. The routine laboratory tests conducted at the time of referral to our hospital were within the normal ranges.Abdominal computed tomography (CT) showed a well-defined, intramural, heterogeneous mass lesion in the gastric body. The mass was oval-shaped and exhibited an enhanced pattern after contrast medium injection. In addition, no enlarged lymph nodes or distant metastasis in other organs around the stomach were observed (Figure 1).In an endoscopic examination of the upper gastrointestinal tract, we observed that the greater curvature of the upper body was covered with relatively normal mucosa. Additionally, a subepithelial lesion approximately 2.5cm in length was observed bulging into the lumen (Figure 2). Using endoscopic ultrasonography (EUS), we observed a round mass of approximately 18mm×12mm in size that originated from the submucosal layer. Within this mass, focal lesions with anechoic foci that appeared to comprise the cystic portion were observed, along with an inhomogeneous pattern (Figures 3A and 3B). On the basis of these combined examination results, the possibility that the lesion was a tumor of mesenchymal origin with malignant potential was explained to the patient and a decision for surgical treatment was made. With the patient under general anesthesia, a laparoscopic wedge resection of the gastric subepithelial lesion was performed. A macroscopic examination revealed a mixed cystic and solid submucosal mass (45mm×22mm×15mm) filled with mucinous material (Figure 4).

**Figure 5:**
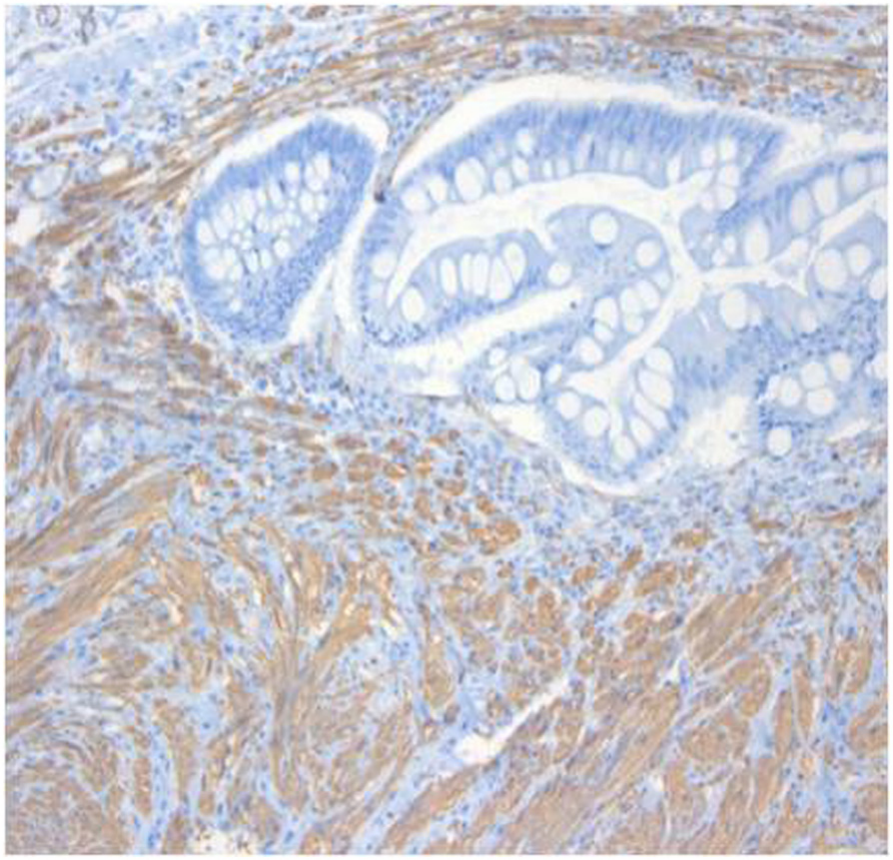
**Immunohistochemical findings.** Smooth muscle actin staining was positive.

Microscopically, broad interdigitating bundles of smooth muscle were present between the ductal collections. Some of the ducts were dilated and lined with tall columnar epithelium featuring regular, basally oriented nuclei. Also some of them were surrounded by branched glands lined with mucus-secreting cells (Figure 4). According to cytologic immunophenotyping, the lesion was consistent with a smooth muscle stromal and epithelial tumor (smooth muscle actin-positive) (Figure 5). The stromal component revealed a low proliferative index (Ki-67 protein immunoexpression, <2%).

The patient did not develop any noticeable complications following the operation. Oral intake was initiated on the third post-operative day, and the patient was discharged from the hospital on the sixth post-operative day. Currently, the patient’s progress is being monitored in the outpatient clinic.

## Discussion

An adenomyoma is a lesion with observed epithelial and smooth muscle proliferation [1,6]. The epithelial origin observed in adenomyomas is unclear, but it is thought that embryonic epithelial buds remaining after birth can develop into undifferentiated glandular structures, mature pancreatic tissues, or duodenal tissues depending on the stage of differentiation. Smooth muscle is thought to accompany the embryonic muscle bud during its separation from the epithelial tissue or to be normal muscle tissue created from proliferating mislocated epithelium consequent to stimulation [7].

The average size of an adenomyoma ranges from 0.6cm to 4.5cm, and these lesions are usually found on the submucosa and sometimes on the muscle layer. This disease can affect patients from 8 months to 82 years of age, and more men are affected than women, albeit this difference is not statistically significant [8,9]. Although gastric adenomyomas can occur anywhere in the gastrointestinal tract, they are most commonly found in the fundus of the gallbladder; most cases in the stomach occur near the antrum or the pylorus [3]. However, in our patient, the lesion was found in the proximal body of the stomach.

Chu described the characteristics of a gastric adenomyoma observed by EUS [10]. However, the pre-surgical diagnosis of this disease remains difficult. In our patient, EUS revealed that the lesion originated from the submucosal layer and contained a cystic center, thus coinciding with previously reported findings [10]. However, because the lesion was located in the proximal body and its cystic center suggested necrotic changes and malignancy, a decision for surgical treatment was made [11].

As previously mentioned, although gastric adenomyomas are characterized as benign tumors, there have been reported cases in which these tumors were found alongside adenocarcinomas. Chapple and colleagues were first to report a case of an adenomyoma observed with adenocarcinoma [2]. Kanehira and colleagues reported a case of an associated adenomyoma with a superficial adenocarcinoma centered in an adenomyoma [12]. Zhu and colleagues demonstrated that frozen sections are useful for intraoperative diagnosis and avoiding unnecessarily extensive surgeries [13]. In our patient, the gastric adenomyoma was confirmed by examining a frozen section taken during the operation, followed by wedge resection of the lesion. The patient’s progress has been observed for 26 months after surgery without any apparent symptoms of recurrence.

## Conclusion

We report that gastric adenomyoma is a very rare disease with differential malignant characteristics that can be observed using EUS. In our patient, we observed that this disease can occur in the stomach body and can be successfully treated with wedge resection of the lesion without a requirement for extensive surgery.

## Consent

Written informed consent was obtained from the patient for publication of this case report and accompanying images. A copy of the written consent is available for review by the Editor-in-Chief of this journal.

## Competing interests

The authors declare that they have no competing interests.

## Authors’ contributions

KHY was a major contributor to the writing of the manuscript. DYE collected and interpreted patient data. JHK and SOL were involved with patient care. HSK was involved with formation of the study concept. DWL carried out the endoscopic examination and contributed to revising the manuscript. All authors read and approved the final manuscript.
